# Supplementary material for: Selective deforestation and exposure of African wildlife to bat-borne viruses
Source: Commun Biol. 2024 Apr 22;7:470. doi: 10.1038/s42003-024-06139-z (PMC11035629; doi:10.1038/s42003-024-06139-z)
Supplement: Supplementary file 2 — Supplementary Information [file 42003_2024_6139_MOESM2_ESM.pdf]

Supplementary material for:

Selective deforestation and exposure of African wildlife to bat-borne viruses

Pawel Fedurek[1,2,†], Caroline Asiimwe[2,†], Gregory K. Rice[3,4], Walter J. Akankwasa[2], Vernon Reynolds[2,5], Catherine Hobaiter[2,6], Robert Kityo[7], Geoffrey Muhanguzi[2], Klaus Zuberbühler[2,6,8], Catherine Crockford[9,10], Regina Z. Cer[3], Andrew J. Bennett[3,4], Jessica M. Rothman[11], Kimberly A. Bishop-Lilly[3], and Tony L. Goldberg[12,*]

[1] Division of Psychology, Faculty of Natural Sciences. University of Stirling, Stirling FK9 4LA, UK
[2] Budongo Conservation Field Station, PO Box 362, Masindi, Uganda
[3] Biological Defense Research Directorate, Naval Medical Research Command, Fort Detrick, MD, 21702, USA
[4] Leidos, 1750 Presidents St, Reston, VA 20190, USA
[5] School of Anthropology, University of Oxford, 51/53 Banbury Road, Oxford OX2 6PE, UK
[6] School of Psychology and Neuroscience, University of St Andrews; St Mary's Quad, South Street, St Andrews KY16 9JP, UK
[7] Department of Zoology, Entomology & Fisheries Sciences, Makerere University, PO Box 7062, Kampala, Uganda
[8] University of Neuchâtel, Department of Comparative Cognition, Avenue du 1er-Mars 26 2000 Neuchâtel, Switzerland
[9] Max Planck Institute for Evolutionary Anthropology, Deutscher Platz 6, 04103 Leipzig, Germany
[10] Institut des Sciences Cognitives, 67 Bd Pinel, 69500 Bron, France
[11] Department of Anthropology, Hunter College of the City University of New York, 695 Park Avenue, New York, NY 10065, USA
[12] Department of Pathobiological Sciences, University of Wisconsin-Madison, 1656 Linden Drive, Madison, WI, USA

† These authors contributed equally

* Correspondence: Tony L. Goldberg (tony.goldberg@wisc.edu)

Contains:

4 Supplementary Tables (1 to 4)

7 Supplementary Figures (1 to 7)

Supplementary Table 1. Viruses identified in bat guano from Budongo Forest, Uganda

	Virus name	Accession	Length (nt)[1]	Genome	Closest match[2]	Family[3]	Genus[3]	Host[4]	% identity[5]
1	Buhirugu virus 1	OP199247	15433	+ssRNA	Bat Hp-betacoronavirus (bat, China, 2013, NC_025217.1)	*Coronaviridae*	*Betacoronavirus*	M	85.75 (99, 0)
2	Buhirugu virus 2	OP834146	6060	+ssRNA	Nilaparvata lugens C virus (planthopper, China, KM270560.1)	*Dicistroviridae*	*Cripavirus*	I	92.66 (99, 0)
3	Buhirugu virus 3	OP834147	5736	+ssRNA	Dicistroviridae sp. (bird anal swab, China, 2018, QJI52207.1)	*Dicistroviridae*	unclassified	I	63.14 (96, 0)
4	Buhirugu virus 4	OP834148	3083	+ssRNA	Nanhai ghost shark hepevirus (shark, China, MG600008.1)	*Hepeviridae*	unclassified	V	45.04 (85, 0)
5	Buhirugu virus 5	OP834149	3039	+ssRNA	Hypnovirus (bat, Republic of the Congo, 2015, MH324434.1)	*Nodaviridae*	unclassified	I,V	81.23 (76, 0)
6	Buhirugu virus 6	OP834150	3072	+ssRNA	Shuangao insect virus 11 (insect, China, 2013, NC_033265.1)	*Nodaviridae*	unclassified	I,V	78.47 (84, 0)
7	Buhirugu virus 7	OP834151	5159	+ssRNA	Tetnovirus 1 (human feces, Afghanistan, 2007, HM480375.1)	unclassified	unclassified	U	64.53 (98, 0)
8	Buhirugu virus 8	OP834152	3027	+ssRNA	Aichivirus A (rat feces, China, 2012, MF352432.1)	*Picornaviridae*	*Kobuvirus*	M	75.54 (96, 0)
9	Buhirugu virus 9	OP834153	6427	+ssRNA	Shanbavirus A (bat, China, 2011, NC_038961.1)	*Picornaviridae*	*Shanbavirus*	U	60.34 (99, 0)
10	Buhirugu virus 10	OP834154	4221	+ssRNA	Acute bee paralysis virus (honeybee, United Kingdom, NC_002548.1)	*Dicistroviridae*	*Aparavirus*	I	30.27 (90, 6E-56)
11	Buhirugu virus 11	OP834155	3228	+ssRNA	Hubei picorna-like virus 23 (spider, China, 2013, KX883692.1)	unclassified	unclassified	I	69.25 (99, 0)
12	Buhirugu virus 12	OP834156	4632	+ssRNA	Violaceae-associated triatovirus 1 (plant, China, MN606293.1)	unclassified	unclassified	P	78.78 (100, 4E-170)
13	Buhirugu virus 13	OP834157	8719	+ssRNA	Solenopsis invicta virus 7 (ant, Argentina, 2014, QBL75890.1)	unclassified	unclassified	I	37.63 (99, 0)
14	Buhirugu virus 14	OP834158	9205	+ssRNA	Picornavirales sp. (bird anal swab, China, 2018, MT138146.1)	*Iflaviridae*	unclassified	I	34.80 (44, 3E-83)
15	Buhirugu virus 15	OP834159	4435	+ssRNA	Niehaus virus (moth, USA, 2016, KX580892.1)	*Permutotetraviridae*	unclassified	I	68.92 (98, 0)
16	Buhirugu virus 16	OP834160	4505	+ssRNA	Daeseongdong virus 2 (mosquito, South Korea, 2012, NC_028489.1)	*Permutotetraviridae*	unclassified	I	47.27 (98, 0)
17	Buhirugu virus 17	OP834161	8178	+ssRNA	Colobopsis shohki virus 1 (ant, Japan, 2016, LC496108.1)	*Polycipiviridae*	*Sopolycivirus*	I	56.26 (98, 0)
18	Buhirugu virus 18	OP834162	1595	dsRNA	Bovine picobirnavirus (cattle, Hong Kong, 2012, KY120179.1)	*Picobirnaviridae*	*Picobirnavirus*	U	72.43 (99, 0)
19	Buhirugu virus 19	OP834163	4177	dsRNA	Bloomfield virus (mouse feces, USA, 2014, MF416371.1)	*Reoviridae*	unclassified	I,V,P	58.39 (99, 0)
20	Buhirugu virus 20	OP834164	2649	dsRNA	Armigeres subalbatus virus (mosquito, China, 2016, NC_014609.1)	*Totiviridae*	unclassified	I	51.51 (85, 0)
21	Buhirugu virus 21	OP834165	2476	dsRNA	Culex tritaeniorhynchus totivirus (mosquito, China, 2010, NC_040670.1)	*Totiviridae*	unclassified	I	64.94 (100, 0)
22	Buhirugu virus 22	OP834166	1269	dsRNA	Hubei diptera virus 19 (dragonfly/damselfly, China, 2013, KX884132.1)	unclassified	unclassified	I	69.36 (99, 0)
23	Buhirugu virus 23	OP834167	1416	dsRNA	Hubei partiti-like virus 41 (dragonfly/damselfly, China, 2013, KX884125.1)	unclassified	unclassified	I	77.28 (100, 0)
24	Buhirugu virus 24	OP834168	1492	dsRNA	Hubei partiti-like virus 40 (insects, China, 2013, KX884195.1)	unclassified	unclassified	I	57.23 (100, 0)
25	Buhirugu virus 25	OP834169	1161	ssDNA	Ambidensovirus sp. (bat, Croatia, 2017, QHY93495.1)	*Parvoviridae*	*Ambidensovirus*	I	79.17 (99, 1E-111)
26	Buhirugu virus 26	OP834170	4824	ssDNA	Bat bocavirus (bat, China, 2013, KX343069.1)	*Parvoviridae*	*Bocaparvovirus*	I,V	67.11 (100, 0)
27	Buhirugu virus 27	OP834171	438	ssDNA	Bat parvovirus (bat, China, 2015, MK757457.1)	*Parvoviridae*	unclassified	M	61.15 (93, 1E-50)

[1] Length in nucleotides of the viral sequence on which comparisons are based
[2] Closest match in the GenBank nucleotide database (source, country, year and accession number of closest match in parentheses, where available)
[3] Family and genus of the closest match in the GenBank database
[4] Typical hosts of viruses in this taxonomic group (I=invertebrate; M=mammal; P=plant; V=vertebrate; U=unknown/unclear)
[5] Percent amino acid sequence identity to the closest match in the GenBank database (query cover and E-value in parentheses)

Supplementary Table 2. Protein sequences used in homology modelling and percent similarity to templates

	ACE2			Aminopeptidase-N			Dipeptidyl peptidase 4			CEACAM1		
**Species**	**Accession**	**PDB ID used as Template**	**Similarity**	**Accession**	**PDB ID used as Template**	**Similarity**	**Accession**	**PDB ID used as Template**	**Similarity**	**Accession**	**PDB ID used as Template**	**Similarity**
BGHRV-1	OP199247[1]	2BEZ	67.5%	OP199247[1]	2BEZ	67.5%	OP199247[1]	2BEZ	67.5%	OP199247[1]	2BEZ	67.5%
Capra hircus	NP_019522936.1	IR42	83.0%	XP_005695088.3	5LG6	84.1%	XP_005676103.1	2QT9	88.1%	KAJ1073265.1	4Y8A	55.6%
Colobus angolensis	XP_011795651.1	IR42	96.3%	XP_011803693.1	5LHD	95.5%	XP_011802485.1	2QT9	98.0%	XP_011791929.1	4QXW	79.4%
Hipposideros armiger	XP_019522936.1	IR42	79.7%	XP_019495554.1	5LHD	79.5%	XP_019492386.1	2QT9	84.3%	XP_019484580.1	4QXW	60.8%
Homo sapiens	Q9BYF1	IR42	100.0%	NP_001368853.1	5LHD	100.0%	NP_001926.2	2QT9	99.9%	P13688	4QXW	100.0%
Pan troglodytes	XP_016798468.1	IR42	99.5%	XP_016782900.1	5LHD	98.6%	XP_515858.2	2QT9	99.5%	H2QGG8	4QXW	96.0%

[1]not a receptor sequence

Supplementary Table 3. MolProbity[1] and Ramachandran scores for BHRGV-1S and modelled putative receptor structures

	ACE2			Aminopeptidase-N			Dipeptidyl peptidase 4			CEACAM1		
**Species**	**MolProbity Score[1]**	**Ramachandran Score (favored region)**	**Ramachandran Outliers**	**MolProbity Score**	**Ramachandran Score (favored region)**	**Ramachandran Outliers**	**MolProbity Score**	**Ramachandran Score (favored region)**	**Ramachandran Outliers**	**MolProbity Score**	**Ramachandran Score (favored region)**	**Ramachandran Outliers**
BGHRV-1	3.05	95.53%	1.97%	3.05	95.53%	1.97%	3.05	95.53%	1.97%	3.05	95.53%	1.97%
Capra hircus	2.40	98.31%	0.34%	1.74	95.68%	0.33%	1.29	95.72%	0.07%	1.67	89.02%	3.20%
Colobus angolensis	2.32	98.15%	0.67%	0.68	97.33%	0.11%	1.25	96.42%	0.14%	1.58	91.20%	3.25%
Hipposideros armiger	2.62	98.32%	0.34%	0.87	97.23%	0.11%	1.39	95.51%	0.14%	1.56	94.46%	0.29%
Homo sapiens	2.49	98.48%	0.34%	1.07	97.34%	0.00%	1.28	96.35%	0.14%	1.49	90.65%	3.63%
Pan troglodytes	2.51	98.65%	0.17%	1.06	97.34%	0.00%	1.26	96.35%	0.14%	1.92	90.31%	3.88%

[1]MolProbity score is a log-weighted combination of the clashscore, percentage Ramachandran not favored and percentage bad side-chain rotamers, giving one number that reflects the crystallographic resolution at which those values would be expected. A value -4 < x < 2 is considered appropriate for normal structures.

Supplementary Table 4. HDOCK docking scores and molecular mechanics Poisson Boltzmann surface area (MM-PBSA) values for modelled binding of BHRGV-1 spike protein to putative receptor molecules of five mammalian species

	**ACE2**		**Aminopeptidase-N**		**Dipeptidyl peptidase 4**		**CEACAM1**	
**Species**	**HDOCK Docking Score**	**MM-PBSA Delta G (kcal/mol)**	**HDOCK Docking Score**	**MM-PBSA Delta G (kcal/mol)**	**HDOCK Docking Score**	**MM-PBSA Delta G (kcal/mol)**	**HDOCK Docking Score**	**MM-PBSA Delta G (kcal/mol)**
Capra hircus	-288.48	-27.7	-275.66	-26.5	-288.45	-27.7	-247.08	-21.8
Colobus angolensis	-308.70	-31.8	-257.34	-24.7	-298.83	-30.7	-258.87	-24.8
Hipposideros armiger	-285.80	-27.4	-284.04	-27.4	-286.06	-27.5	-288.68	-27.7
Homo sapiens	-302.94	-30.7	-271.59	-26.2	-304.50	-31.1	-294.30	-29.5
Pan troglodytes	-309.14	-32.2	-271.67	-26.2	-319.39	-35.3	-265.82	-26.9

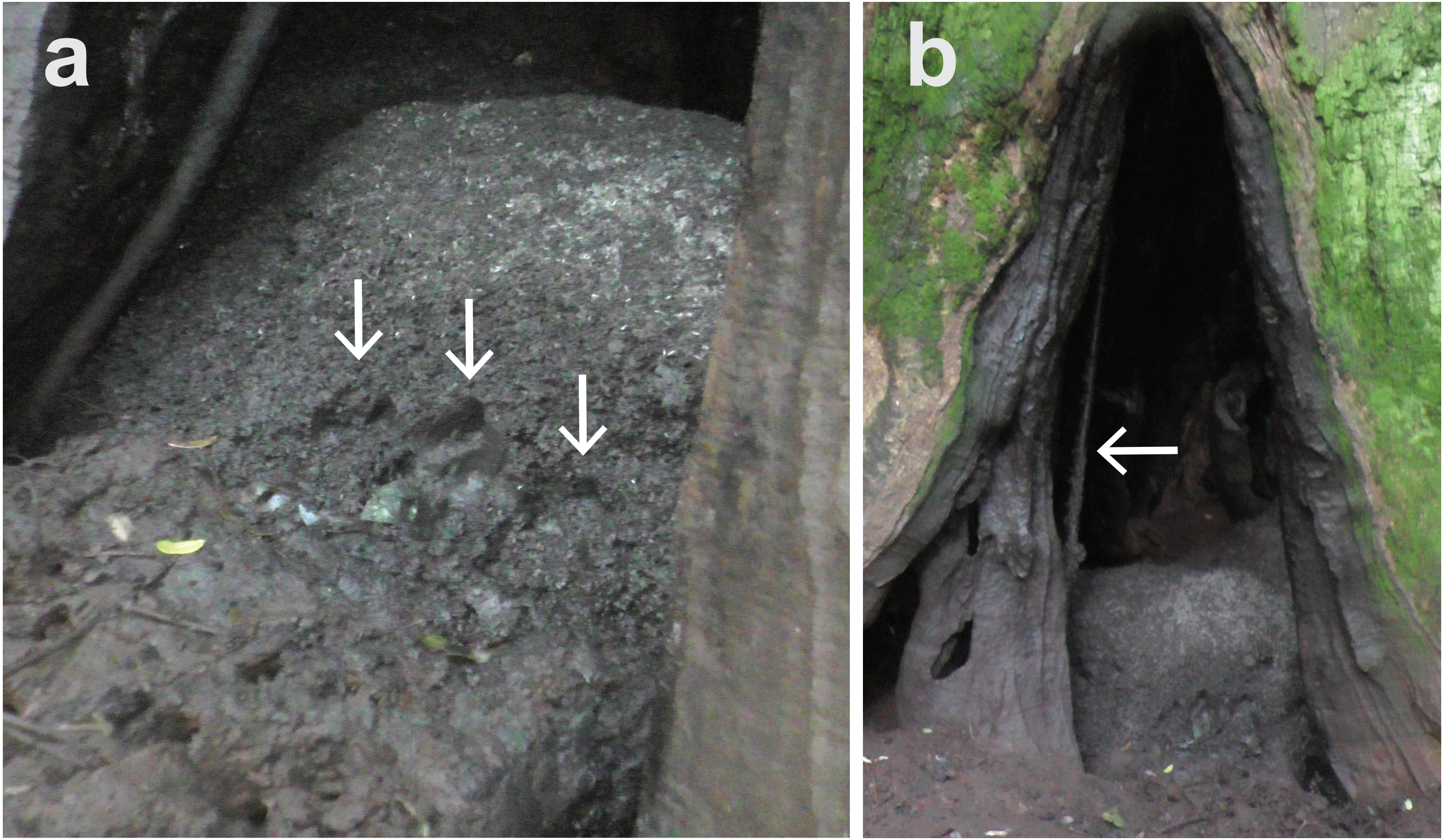

Supplementary Figure 1. Images of (**a**) excavations and chimpanzee hand prints in the bat guano pile (arrows), and (**b**) a human-modified pole (arrow) inside the hollow tree containing the guano pile.

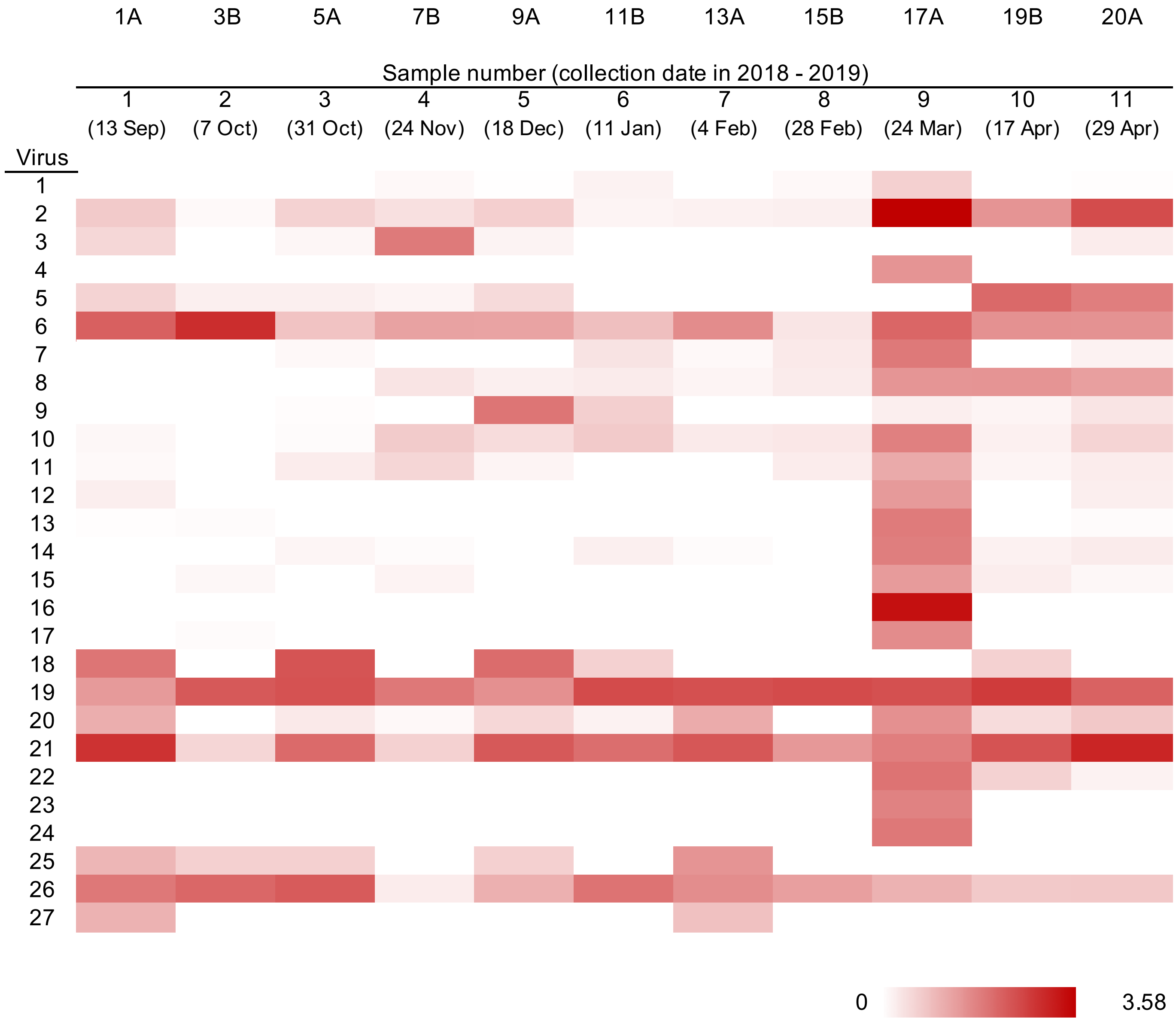

Supplementary Figure 2. Heatmap of viral concentrations in 11 bat guano samples from Budongo Forest, Uganda, collected between September 13, 2018 and April 29, 2019. Cells are shaded in proportion to log10 (viral reads per 10^6 total reads per kilobase of target sequence + 1). Virus numbers refer to Supplementary Table 1.

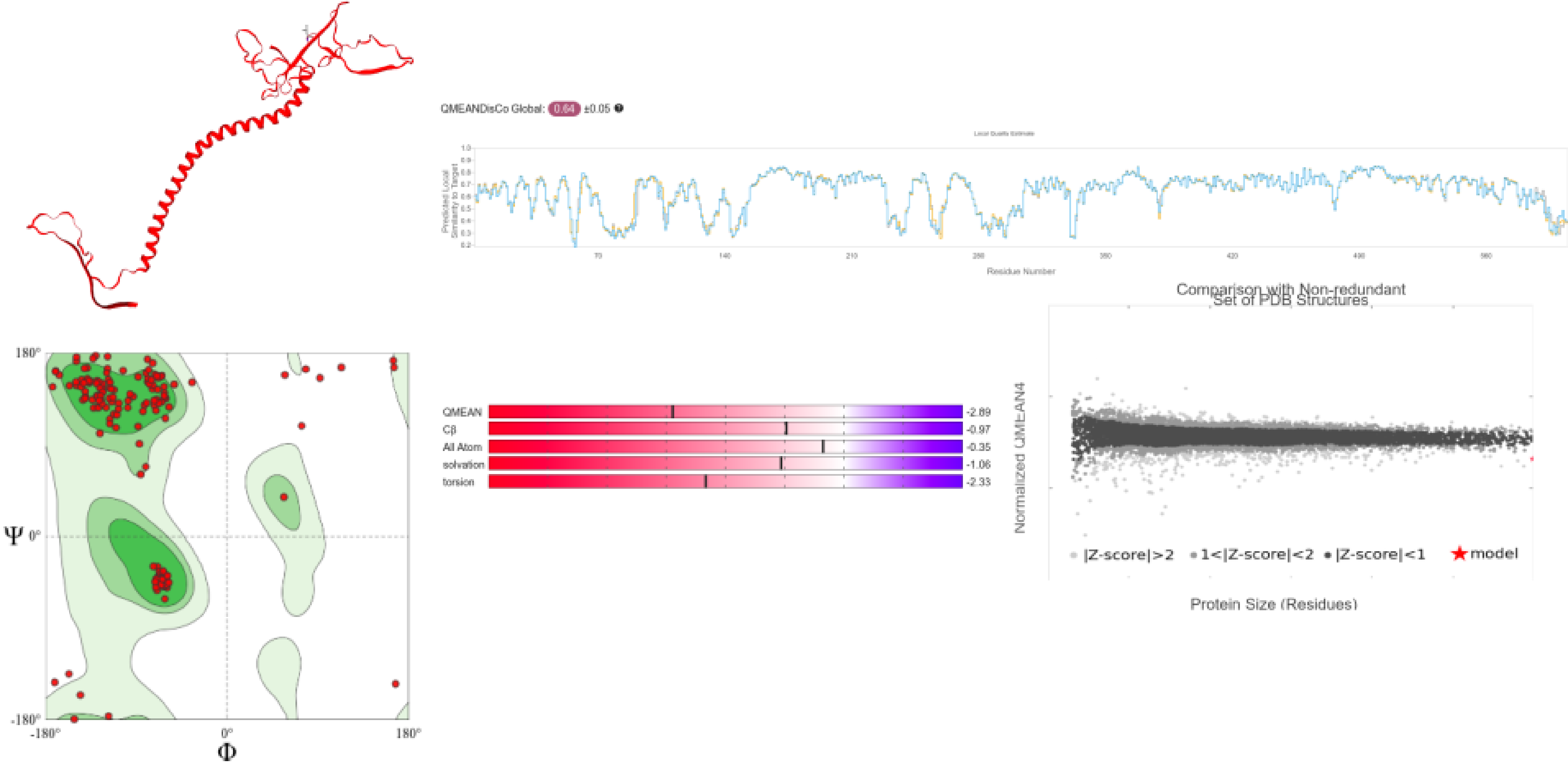

Supplementary Figure 3. Modelled BGHRV-1 spike protein structure together with Ramachandran plot and quality metrics.

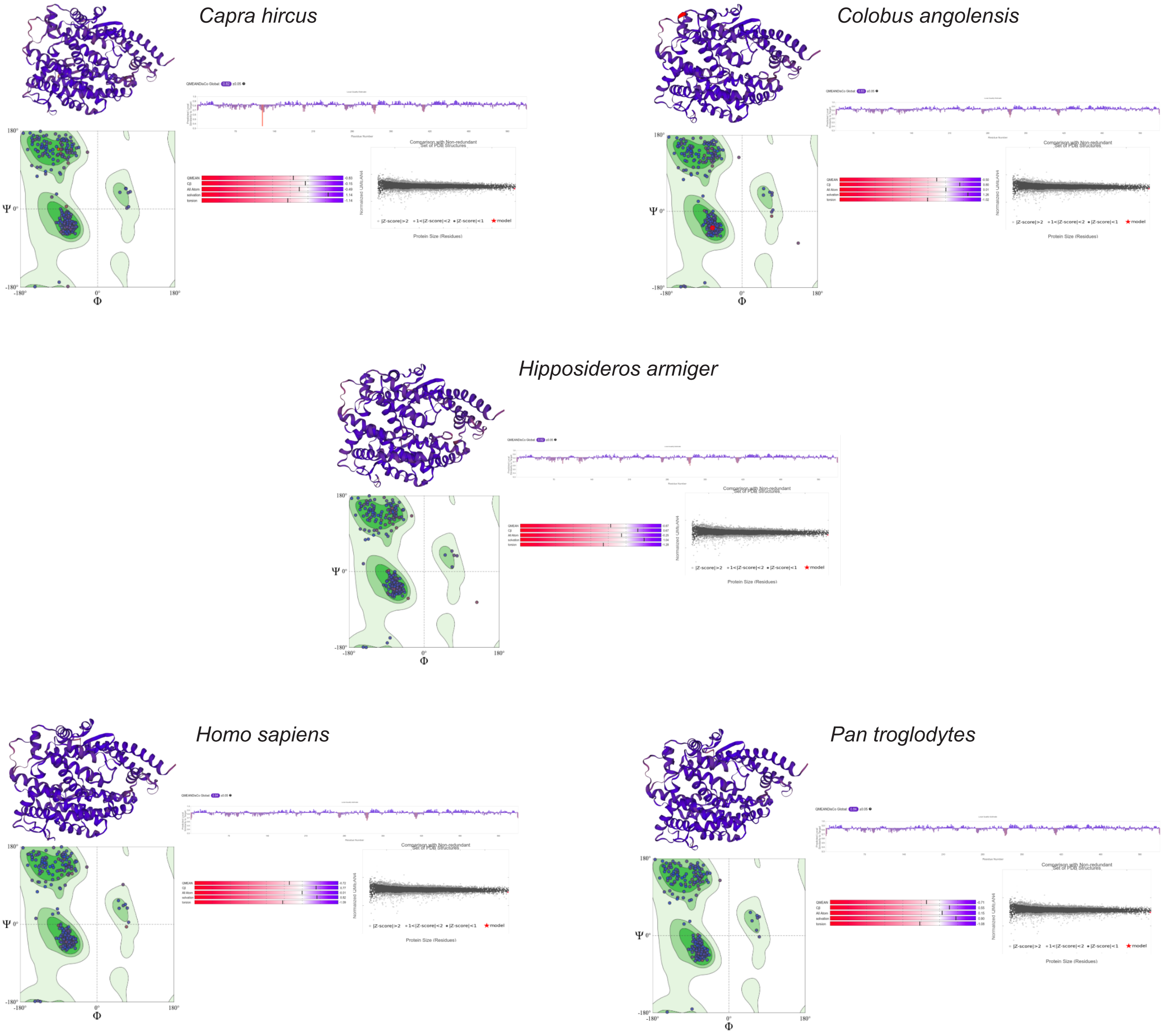

Supplementary Figure 4. Modelled ACE2 receptor structure together with Ramachandran plot and quality metrics for *Capra hircus*, *Colobus angolensis palliates*, *Hipposideros armiger*, *Homo sapiens*, and *Pan troglodytes*.

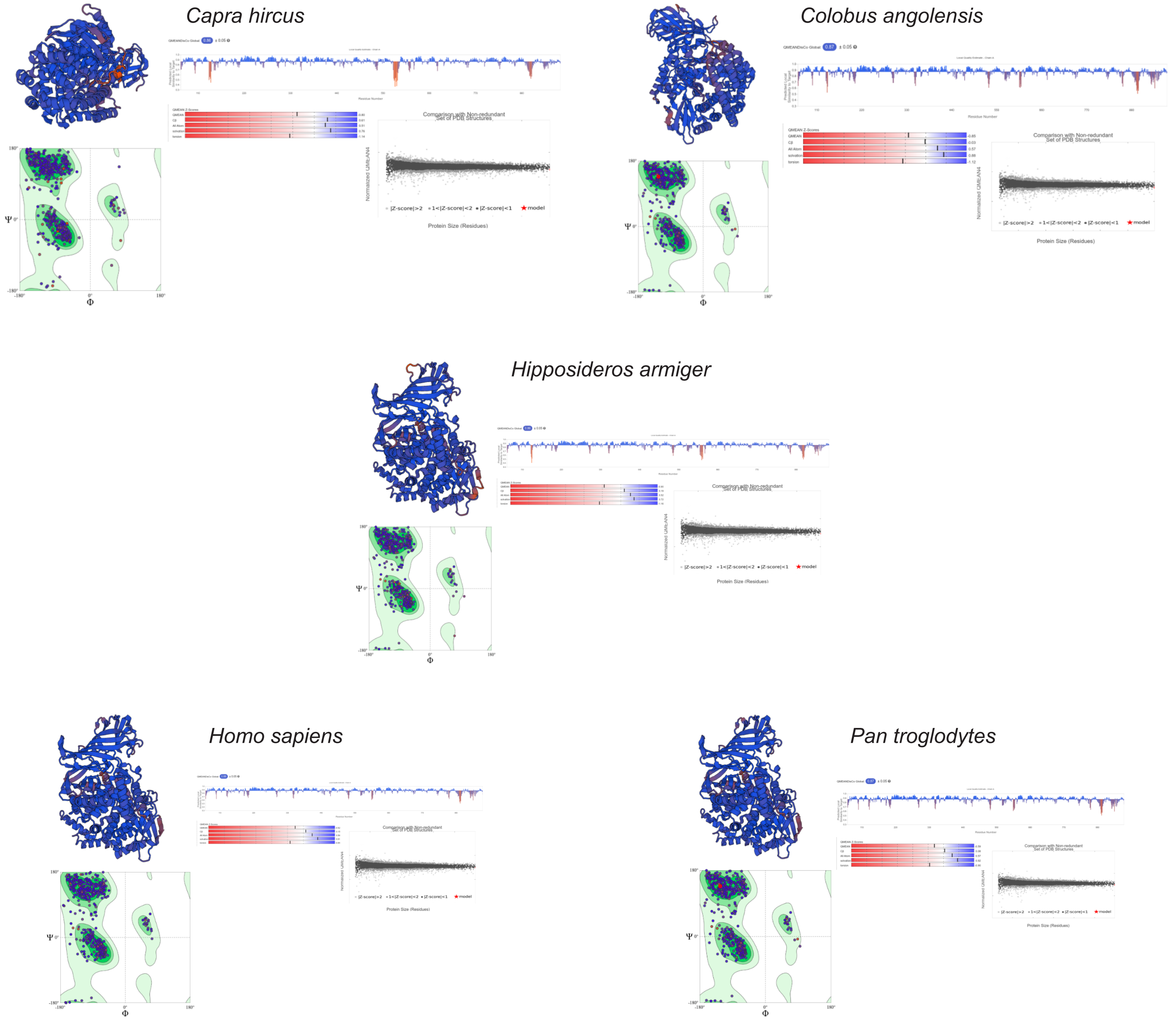

Supplementary Figure 5. Modelled Aminopeptidase-N receptor structure together with Ramachandran plot and quality metrics for *Capra hircus*, *Colobus angolensis palliates*, *Hipposideros armiger*, *Homo sapiens*, and *Pan troglodytes*.

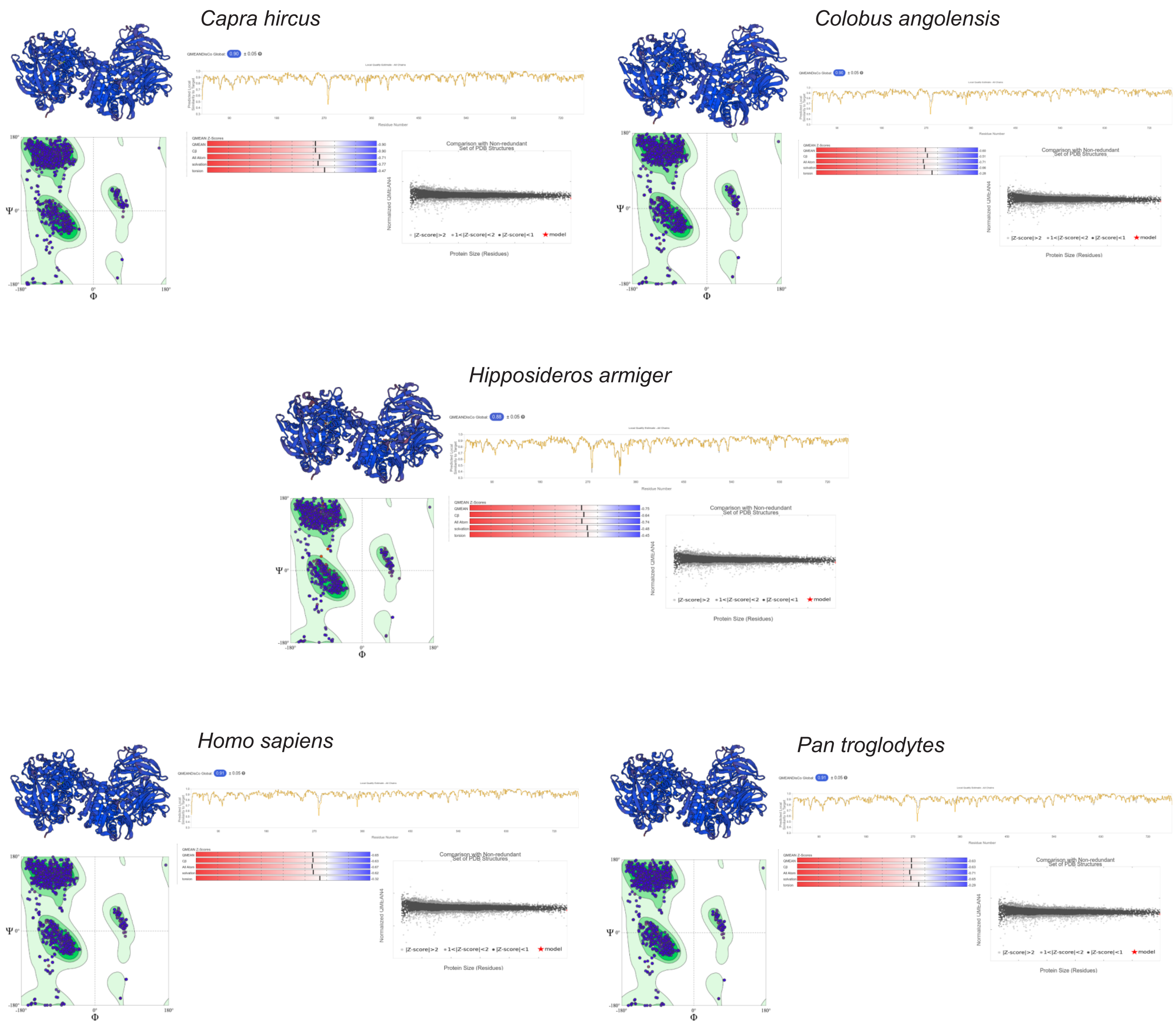

Supplementary Figure 6. Modelled Dipeptidyl peptidase 4 receptor structure together with Ramachandran plot and quality metrics for *Capra hircus*, *Colobus angolensis palliates*, *Hipposideros armiger*, *Homo sapiens*, and *Pan troglodytes*.

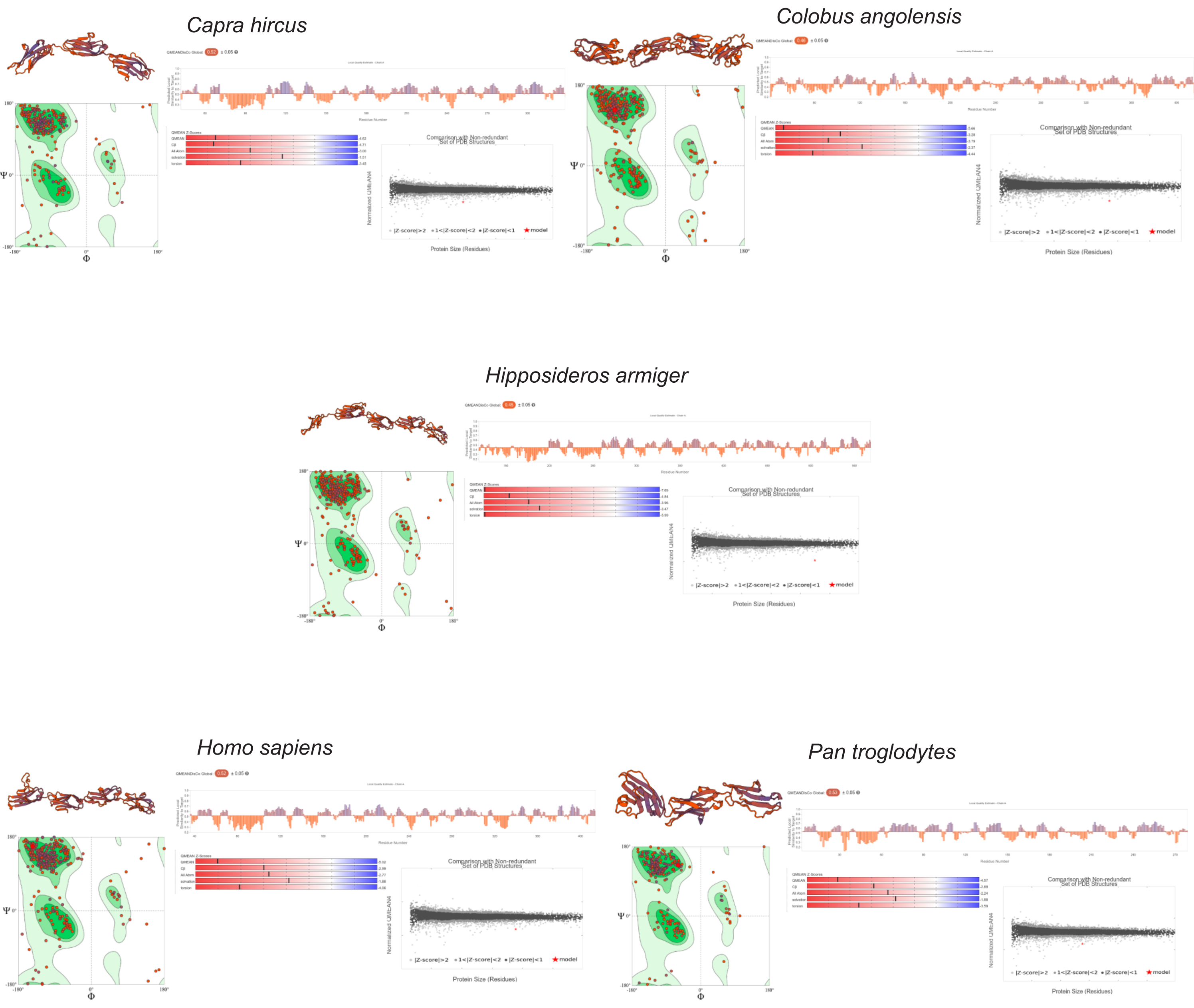

Supplementary Figure 7. Modelled CEACAM1 receptor structure together with Ramachandran plot and quality metrics for *Capra hircus*, *Colobus angolensis palliates*, *Hipposideros armiger*, *Homo sapiens*, and *Pan troglodytes*.
