# Supplementary material for: Selective deforestation and exposure of African wildlife to bat-borne viruses
Source: Commun Biol. 2024 Apr 22;7:470. doi: 10.1038/s42003-024-06139-z (PMC11035629; doi:10.1038/s42003-024-06139-z)
Supplement: Supplementary file 3 — Description of Additional Supplementary Files [file 42003_2024_6139_MOESM3_ESM.pdf]

Description of Additional Supplementary Files

File name: Supplementary Video 1

Description: Video of chimpanzees consuming guano

File name: Supplementary Video 2

Description: Video of chimpanzees consuming guano

File name: Supplementary Video 3

Description: Video of chimpanzees drinking water adjacent to guano using a leaf sponge

File name: Supplementary Video 4

Description: Video of black-and-white colobus consuming guano

File name: Supplementary Video 5

Description: Video of red duiker consuming guano
